# Aqua­{*N*,*N*-dimethyl-*N*′-[1-(2-pyrid­yl)ethyl­idene]ethane-1,2-diamine-κ^3^ *N*,*N*′,*N*′′}bis­(thio­cyanato-κ*N*)nickel(II)

**DOI:** 10.1107/S1600536811011512

**Published:** 2011-03-31

**Authors:** Nura Suleiman Gwaram, Siti Munirah Saharin, Hamid Khaledi, Hapipah Mohd Ali

**Affiliations:** a Department of Chemistry, University of Malaya, 50603 Kuala Lumpur, Malaysia

## Abstract

In the title compound, [Ni(NCS)_2_(C_11_H_17_N_3_)(H_2_O)], the Ni^II^ ion is six-coordinated by the *N,N′,N"*-tridentate Schiff base N atoms, two *cis*-positioned *N*-bound isothio­cyanate groups and one water mol­ecule. In the crystal, O—H⋯S hydrogen bonds link adjacent mol­ecules into infinite layers parallel to the *ac* plane. The layers are further connected into a three-dimensional network *via* C—H⋯π inter­actions. The –CH_2_–N(CH_3_)_2_ fragment is disordered over two sets of sites in a 0.556 (5):0.444 (5) ratio.

## Related literature

For the structure of a similar mononuclear nickel(II) thio­cyanate complex, see: Suleiman Gwaram *et al.* (2011 ▶). For dimeric nickel(II) thio­cyanate complexes with similar Schiff bases, see: Diao (2007 ▶); Bhowmik *et al.* (2010 ▶).
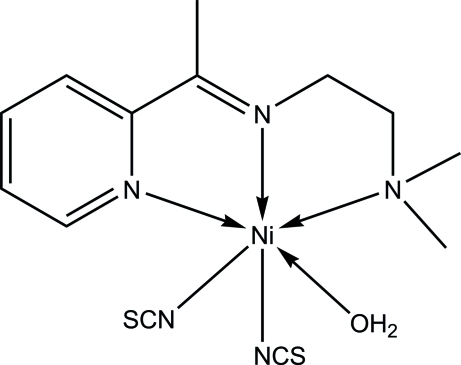


## Experimental

### 

#### Crystal data

[Ni(NCS)_2_(C_11_H_17_N_3_)(H_2_O)]
*M* *_r_* = 384.16
Monoclinic, 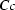

*a* = 12.8404 (2) Å
*b* = 14.2623 (3) Å
*c* = 9.5868 (2) Å
β = 99.467 (1)°
*V* = 1731.75 (6) Å^3^
*Z* = 4
Mo *K*α radiation
μ = 1.37 mm^−1^
*T* = 100 K
0.22 × 0.19 × 0.11 mm

#### Data collection

Bruker APEXII CCD diffractometer
Absorption correction: multi-scan (*SADABS*; Sheldrick, 1996 ▶) *T*_min_ = 0.753, *T*_max_ = 0.864
7792 measured reflections
3698 independent reflections
3451 reflections with *I* > 2σ(*I*)
*R*_int_ = 0.026

#### Refinement

*R*[*F* ^2^ > 2σ(*F* ^2^)] = 0.031
*wR*(*F* ^2^) = 0.070
*S* = 1.02
3698 reflections
234 parameters
16 restraints
H atoms treated by a mixture of independent and constrained refinement
Δρ_max_ = 0.58 e Å^−3^
Δρ_min_ = −0.52 e Å^−3^
Absolute structure: Flack (1983 ▶), 1798 Friedel pairs
Flack parameter: 0.020 (11)

### 

Data collection: *APEX2* (Bruker, 2007 ▶); cell refinement: *SAINT* (Bruker, 2007 ▶); data reduction: *SAINT*; program(s) used to solve structure: *SHELXS97* (Sheldrick, 2008 ▶); program(s) used to refine structure: *SHELXL97* (Sheldrick, 2008 ▶); molecular graphics: *X-SEED* (Barbour, 2001 ▶); software used to prepare material for publication: *SHELXL97* and *publCIF* (Westrip, 2010 ▶).

## Supplementary Material

Crystal structure: contains datablocks I, global. DOI: 10.1107/S1600536811011512/go2008sup1.cif

Structure factors: contains datablocks I. DOI: 10.1107/S1600536811011512/go2008Isup2.hkl

Additional supplementary materials:  crystallographic information; 3D view; checkCIF report

## Figures and Tables

**Table 1:** Hydrogen-bond geometry (Å, °) *Cg*1 is the centroid of the N1,C1–C5 ring.

*D*—H⋯*A*	*D*—H	H⋯*A*	*D*⋯*A*	*D*—H⋯*A*
O1—H1*B*⋯S1^i^	0.82 (2)	2.38 (2)	3.181 (3)	164 (4)
O1—H1*A*⋯S2^ii^	0.84 (2)	2.35 (2)	3.190 (3)	178 (4)
C7—H7*C*⋯*Cg*1^iii^	0.98	2.88	3.531 (3)	125

Symmetry codes: (i) 
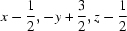
; (ii) 
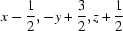
; (iii) 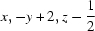.

**Fig. 1.**
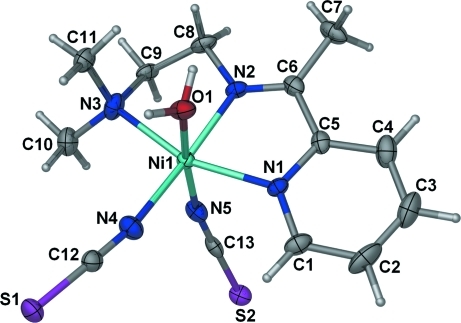
Thermal ellipsoid plot of the title compound at the 50% probability level. Hydrogen atoms are drawn as spheres of arbitrary radius. Only the major disordered component is shown.

**Fig. 2.**
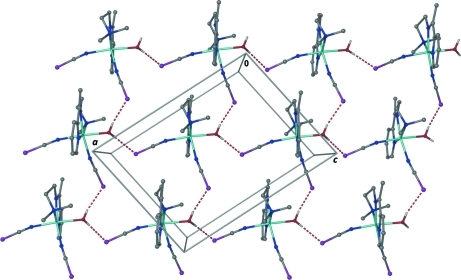
Unit-cell packing of the title compound, viewed down the b axis, showing the O—H···S hydrogen-bonded two-dimensional network. C-bound hydrogen atoms have been omitted for clarity.

## supplementary crystallographic information

### Comment

The title mixed-ligand complex was obtained *via* the treatment of nickel(II) ion with the Schiff base *N,N*-dimethyl-*N'*-[methyl(2-pyridyl)methylene]ethane-1,2-diamine, prepared *in situ*, and the thiocyanate salt. The Schiff base acts as an *N,N',N"*-tridentate chelate and the two thiocyanate ions behave in an *N*-donor fashion towards the Ni^II^ ion. The geometry around the metal center is completed by one water O atom. This arrangement is similar to what was observed in the nickel(II) thiocyanate complex of a similar Schiff base (Suleiman Gwaram *et al.*, 2011). In contrast, the metal ions in the nickel(II) thiocyanate complex of *N,N*-dimethyl-*N'*-(2-pyridylmethylene)ethane-1,2-diamine (Diao, 2007) and *N,N*-diethyl-*N'*-[methyl(2-pyridyl)methylene]ethane-1,2-diamine (Bhowmik *et al.*, 2010) are doubly bridged into dimers by *N:S*-bridging thiocyanate ligands. In the present structure, the adjacent molecules are connected into 2-D arrays in *ac* plane *via* O—H···S interactions (Table 1, Fig. 2). A C—H···π interaction (Table 1) connects the layers into a three-dimensional structure.

### Experimental

A mixture of 2-acetylpyridine (0.2 g, 1.65 mmol) and *N,N*-dimethylethyldiamine (0.15 g, 1.65 mmol) in ethanol (20 ml) was refluxed for 2 h followed by addition of a solution of nickel(II) acetate tetrahydrate (0.41 g, 1.65 mmol) and sodium thiocyanate (0.27 g, 3.3 mmol) in a minimum amount of water. The resulting solution was refluxed for 30 min, then set aside at room temperature. Brown crystals of the title compound were obtained by slow evaporation of the resulting reaction mixture.

### Refinement

The C-bound H atoms were placed at calculated positions at distances C—H = 0.95, 0.98 and 0.99 Å for aryl, methyl and methylene type H-atoms, respectively. The O-bound H atoms were placed in a difference Fourier map, and were refined with distance restraint of O—H 0.84 (2) Å. For all hydrogen atoms U*iso*(H) were set to 1.2–1.5 times U*eq*(carrier atom). C9, C10 and C11 were found to be disordered with two positions being resolved for each of the atoms. From anisotropic refinement, the major component of the disorder had a site occupancy factor of 0.556 (5). The N3—C_methyl_ bond distances were restrained to be 1.470±0.001 Å. The N3—C9 and N3—C9' bond distances were refined with the distance restraint of 1.480±0.001 Å. The C8—C9 and C8—C9' bond distances were refined with the distance restraint of 1.52±0.001 Å. The corresponding bond distances involving the disordered atoms were restrained to be equal with the SADI command in *SHELXL97* (Sheldrick, 2008). An absolute structure was established using anomalous dispersion effects; 1798 Friedel pairs were not merged.

### Crystal data

**Table:**

[Ni(NCS)_2_(C_11_H_17_N_3_)(H_2_O)]	*F*(000) = 800
*M**_r_* = 384.16	*D*_x_ = 1.473 Mg m^−^^3^
Monoclinic, *C**c*	Mo *K*α radiation, λ = 0.71073 Å
Hall symbol: C -2yc	Cell parameters from 2239 reflections
*a* = 12.8404 (2) Å	θ = 2.9–27.9°
*b* = 14.2623 (3) Å	µ = 1.37 mm^−^^1^
*c* = 9.5868 (2) Å	*T* = 100 K
β = 99.467 (1)°	Block, brown
*V* = 1731.75 (6) Å^3^	0.22 × 0.19 × 0.11 mm
*Z* = 4	

### Data collection

**Table:**

Bruker APEXII CCD diffractometer	3698 independent reflections
Radiation source: fine-focus sealed tube	3451 reflections with *I* > 2σ(*I*)
graphite	*R*_int_ = 0.026
φ and ω scans	θ_max_ = 27.0°, θ_min_ = 2.2°
Absorption correction: multi-scan (*SADABS*; Sheldrick, 1996)	*h* = −16→16
*T*_min_ = 0.753, *T*_max_ = 0.864	*k* = −18→18
7792 measured reflections	*l* = −12→12

### Refinement

**Table:**

Refinement on *F*^2^	Secondary atom site location: difference Fourier map
Least-squares matrix: full	Hydrogen site location: inferred from neighbouring sites
*R*[*F*^2^ > 2σ(*F*^2^)] = 0.031	H atoms treated by a mixture of independent and constrained refinement
*wR*(*F*^2^) = 0.070	*w* = 1/[σ^2^(*F*_o_^2^) + (0.0313*P*)^2^] where *P* = (*F*_o_^2^ + 2*F*_c_^2^)/3
*S* = 1.02	(Δ/σ)_max_ = 0.001
3698 reflections	Δρ_max_ = 0.58 e Å^−^^3^
234 parameters	Δρ_min_ = −0.52 e Å^−^^3^
16 restraints	Absolute structure: Flack (1983), 1798 Friedel pairs
Primary atom site location: structure-invariant direct methods	Flack parameter: 0.020 (11)

### Special details

**Table:**

Geometry. All e.s.d.'s (except the e.s.d. in the dihedral angle between two l.s. planes) are estimated using the full covariance matrix. The cell e.s.d.'s are taken into account individually in the estimation of e.s.d.'s in distances, angles and torsion angles; correlations between e.s.d.'s in cell parameters are only used when they are defined by crystal symmetry. An approximate (isotropic) treatment of cell e.s.d.'s is used for estimating e.s.d.'s involving l.s. planes.
Refinement. Refinement of *F*^2^ against ALL reflections. The weighted *R*-factor *wR* and goodness of fit *S* are based on *F*^2^, conventional *R*-factors *R* are based on *F*, with *F* set to zero for negative *F*^2^. The threshold expression of *F*^2^ > σ(*F*^2^) is used only for calculating *R*-factors(gt) *etc*. and is not relevant to the choice of reflections for refinement. *R*-factors based on *F*^2^ are statistically about twice as large as those based on *F*, and *R*- factors based on ALL data will be even larger.

### Fractional atomic coordinates and isotropic or equivalent isotropic displacement parameters (Å^2^)

**Table:**

	*x*	*y*	*z*	*U*_iso_*/*U*_eq_	Occ. (<1)
Ni1	0.53275 (7)	0.77376 (2)	0.26546 (8)	0.01875 (9)	
S1	0.66956 (9)	0.64265 (6)	0.72084 (11)	0.02834 (19)	
S2	0.87020 (9)	0.82690 (6)	0.13658 (11)	0.02889 (19)	
O1	0.3999 (2)	0.79724 (19)	0.3691 (3)	0.0272 (5)	
H1A	0.392 (3)	0.763 (2)	0.438 (3)	0.041*	
H1B	0.3417 (19)	0.804 (3)	0.319 (3)	0.041*	
N1	0.55528 (19)	0.91914 (17)	0.2861 (3)	0.0203 (6)	
N2	0.4415 (2)	0.82344 (18)	0.0894 (2)	0.0212 (5)	
N3	0.47431 (16)	0.64117 (17)	0.1766 (2)	0.0310 (6)	
N4	0.6034 (2)	0.72811 (19)	0.4589 (3)	0.0292 (6)	
N5	0.6702 (3)	0.7681 (2)	0.1831 (3)	0.0278 (7)	
C1	0.6229 (3)	0.9645 (2)	0.3838 (3)	0.0286 (7)	
H1	0.6663	0.9295	0.4551	0.034*	
C2	0.6315 (3)	1.0619 (3)	0.3840 (4)	0.0433 (10)	
H2	0.6805	1.0929	0.4540	0.052*	
C3	0.5683 (3)	1.1122 (3)	0.2819 (4)	0.0421 (10)	
H3	0.5722	1.1788	0.2816	0.050*	
C4	0.4990 (3)	1.0663 (2)	0.1793 (4)	0.0360 (8)	
H4	0.4552	1.1004	0.1070	0.043*	
C5	0.4946 (2)	0.9689 (2)	0.1842 (3)	0.0223 (6)	
C6	0.4261 (2)	0.9115 (2)	0.0758 (3)	0.0242 (6)	
C7	0.3465 (3)	0.9581 (3)	−0.0336 (3)	0.0360 (8)	
H7A	0.2898	0.9845	0.0114	0.054*	
H7B	0.3168	0.9119	−0.1049	0.054*	
H7C	0.3806	1.0084	−0.0791	0.054*	
C8	0.3855 (3)	0.7537 (2)	−0.0046 (3)	0.0297 (7)	
H8A	0.3824	0.7729	−0.1044	0.036*	
H8B	0.3125	0.7462	0.0144	0.036*	
C9	0.4462 (4)	0.6625 (3)	0.0238 (2)	0.0265 (14)	0.556 (5)
H9A	0.4029	0.6106	−0.0234	0.032*	0.556 (5)
H9B	0.5116	0.6663	−0.0179	0.032*	0.556 (5)
C10	0.5496 (4)	0.5631 (3)	0.2016 (7)	0.0375 (17)	0.556 (5)
H10A	0.5166	0.5060	0.1578	0.056*	0.556 (5)
H10B	0.5700	0.5532	0.3035	0.056*	0.556 (5)
H10C	0.6124	0.5780	0.1600	0.056*	0.556 (5)
C11	0.3747 (3)	0.6125 (4)	0.2213 (6)	0.0306 (15)	0.556 (5)
H11A	0.3520	0.5521	0.1780	0.046*	0.556 (5)
H11B	0.3203	0.6599	0.1912	0.046*	0.556 (5)
H11C	0.3854	0.6065	0.3245	0.046*	0.556 (5)
C9'	0.3761 (3)	0.6644 (4)	0.0790 (7)	0.045 (2)	0.444 (5)
H9'A	0.3179	0.6716	0.1342	0.053*	0.444 (5)
H9'B	0.3579	0.6117	0.0122	0.053*	0.444 (5)
C10'	0.5540 (5)	0.5991 (6)	0.1017 (9)	0.045 (2)	0.444 (5)
H10D	0.5281	0.5388	0.0610	0.067*	0.444 (5)
H10E	0.6197	0.5893	0.1682	0.067*	0.444 (5)
H10F	0.5675	0.6412	0.0259	0.067*	0.444 (5)
C11'	0.4673 (8)	0.5708 (4)	0.2867 (6)	0.039 (2)	0.444 (5)
H11D	0.4406	0.5117	0.2424	0.059*	0.444 (5)
H11E	0.4191	0.5932	0.3489	0.059*	0.444 (5)
H11F	0.5375	0.5606	0.3425	0.059*	0.444 (5)
C12	0.6312 (3)	0.6935 (2)	0.5665 (3)	0.0236 (6)	
C13	0.7532 (3)	0.7906 (2)	0.1624 (3)	0.0244 (7)	

### Atomic displacement parameters (Å^2^)

**Table:**

	*U*^11^	*U*^22^	*U*^33^	*U*^12^	*U*^13^	*U*^23^
Ni1	0.02124 (18)	0.01989 (18)	0.01472 (16)	−0.00167 (17)	0.00176 (13)	0.00004 (16)
S1	0.0305 (5)	0.0303 (4)	0.0227 (4)	0.0010 (3)	−0.0001 (3)	0.0074 (3)
S2	0.0281 (4)	0.0372 (5)	0.0227 (4)	0.0023 (3)	0.0082 (3)	0.0004 (3)
O1	0.0213 (13)	0.0379 (14)	0.0219 (12)	−0.0058 (10)	0.0023 (10)	0.0021 (10)
N1	0.0196 (15)	0.0235 (13)	0.0185 (13)	−0.0052 (10)	0.0055 (11)	−0.0028 (10)
N2	0.0195 (13)	0.0293 (15)	0.0150 (12)	−0.0046 (11)	0.0030 (10)	−0.0051 (10)
N3	0.0463 (18)	0.0230 (15)	0.0254 (13)	−0.0079 (12)	0.0112 (13)	−0.0063 (11)
N4	0.0311 (16)	0.0345 (17)	0.0222 (14)	0.0040 (12)	0.0044 (12)	0.0035 (12)
N5	0.0287 (17)	0.0344 (17)	0.0215 (14)	0.0071 (13)	0.0075 (13)	0.0040 (11)
C1	0.0273 (18)	0.037 (2)	0.0227 (16)	−0.0095 (15)	0.0072 (14)	−0.0096 (14)
C2	0.049 (2)	0.046 (2)	0.039 (2)	−0.0252 (19)	0.0200 (19)	−0.0243 (18)
C3	0.063 (3)	0.0250 (18)	0.045 (2)	−0.0114 (16)	0.027 (2)	−0.0077 (16)
C4	0.052 (2)	0.0250 (19)	0.0353 (18)	0.0013 (16)	0.0211 (18)	0.0043 (14)
C5	0.0256 (17)	0.0223 (16)	0.0217 (14)	0.0011 (13)	0.0117 (13)	0.0036 (12)
C6	0.0230 (16)	0.0302 (18)	0.0206 (15)	0.0014 (13)	0.0073 (12)	0.0066 (13)
C7	0.0299 (18)	0.051 (2)	0.0275 (18)	0.0088 (16)	0.0050 (15)	0.0158 (16)
C8	0.0271 (17)	0.042 (2)	0.0194 (15)	−0.0122 (15)	0.0010 (13)	−0.0066 (13)
C9	0.023 (3)	0.027 (3)	0.029 (3)	−0.004 (2)	0.001 (3)	−0.012 (2)
C10	0.050 (4)	0.027 (4)	0.037 (4)	0.001 (3)	0.013 (3)	0.002 (3)
C11	0.035 (3)	0.023 (3)	0.034 (3)	−0.005 (2)	0.007 (3)	−0.005 (2)
C9'	0.035 (5)	0.050 (6)	0.050 (5)	−0.018 (4)	0.012 (4)	−0.021 (4)
C10'	0.039 (5)	0.025 (5)	0.065 (6)	0.000 (4)	−0.008 (5)	−0.007 (4)
C11'	0.063 (6)	0.017 (4)	0.039 (5)	0.001 (4)	0.013 (4)	−0.003 (3)
C12	0.0241 (16)	0.0217 (16)	0.0249 (16)	0.0005 (13)	0.0037 (13)	−0.0018 (13)
C13	0.0317 (18)	0.0262 (17)	0.0160 (14)	0.0121 (14)	0.0060 (13)	0.0054 (12)

### Geometric parameters (Å, °)

**Table:**

Ni1—N2	2.018 (2)	C4—C5	1.392 (4)
Ni1—N4	2.033 (3)	C4—H4	0.9500
Ni1—N5	2.050 (3)	C5—C6	1.491 (4)
Ni1—N1	2.098 (2)	C6—C7	1.495 (4)
Ni1—O1	2.137 (2)	C7—H7A	0.9800
Ni1—N3	2.158 (2)	C7—H7B	0.9800
S1—C12	1.648 (3)	C7—H7C	0.9800
S2—C13	1.645 (4)	C8—C9	1.5180 (10)
O1—H1A	0.836 (18)	C8—C9'	1.5204 (10)
O1—H1B	0.824 (19)	C8—H8A	0.9900
N1—C1	1.335 (4)	C8—H8B	0.9900
N1—C5	1.347 (4)	C9—H9A	0.9900
N2—C6	1.275 (4)	C9—H9B	0.9900
N2—C8	1.451 (4)	C10—H10A	0.9800
N3—C10	1.4683 (10)	C10—H10B	0.9800
N3—C11'	1.4706 (10)	C10—H10C	0.9800
N3—C10'	1.4716 (10)	C11—H11A	0.9800
N3—C11	1.4725 (10)	C11—H11B	0.9800
N3—C9'	1.4783 (10)	C11—H11C	0.9800
N3—C9	1.4810 (10)	C9'—H9'A	0.9900
N4—C12	1.147 (4)	C9'—H9'B	0.9900
N5—C13	1.162 (4)	C10'—H10D	0.9800
C1—C2	1.394 (5)	C10'—H10E	0.9800
C1—H1	0.9500	C10'—H10F	0.9800
C2—C3	1.368 (5)	C11'—H11D	0.9800
C2—H2	0.9500	C11'—H11E	0.9800
C3—C4	1.378 (5)	C11'—H11F	0.9800
C3—H3	0.9500		
			
N2—Ni1—N4	170.38 (10)	N1—C5—C6	114.9 (3)
N2—Ni1—N5	96.32 (11)	C4—C5—C6	123.1 (3)
N4—Ni1—N5	93.17 (11)	N2—C6—C5	113.9 (3)
N2—Ni1—N1	77.53 (9)	N2—C6—C7	125.9 (3)
N4—Ni1—N1	101.33 (10)	C5—C6—C7	120.2 (3)
N5—Ni1—N1	87.79 (10)	C6—C7—H7A	109.5
N2—Ni1—O1	86.32 (9)	C6—C7—H7B	109.5
N4—Ni1—O1	84.06 (10)	H7A—C7—H7B	109.5
N5—Ni1—O1	171.42 (11)	C6—C7—H7C	109.5
N1—Ni1—O1	84.80 (10)	H7A—C7—H7C	109.5
N2—Ni1—N3	82.01 (10)	H7B—C7—H7C	109.5
N4—Ni1—N3	98.83 (10)	N2—C8—C9	106.8 (3)
N5—Ni1—N3	94.52 (11)	N2—C8—C9'	108.7 (3)
N1—Ni1—N3	159.55 (9)	N2—C8—H8A	110.4
O1—Ni1—N3	93.93 (10)	C9—C8—H8A	110.4
Ni1—O1—H1A	119 (3)	C9'—C8—H8A	138.3
Ni1—O1—H1B	118 (3)	N2—C8—H8B	110.4
H1A—O1—H1B	108 (4)	C9—C8—H8B	110.4
C1—N1—C5	119.0 (3)	C9'—C8—H8B	69.7
C1—N1—Ni1	127.5 (2)	H8A—C8—H8B	108.6
C5—N1—Ni1	113.44 (19)	N3—C9—C8	112.8 (3)
C6—N2—C8	124.0 (3)	N3—C9—H9A	109.0
C6—N2—Ni1	119.4 (2)	C8—C9—H9A	109.0
C8—N2—Ni1	116.04 (19)	N3—C9—H9B	109.0
C10—N3—C11'	58.8 (4)	C8—C9—H9B	109.0
C11'—N3—C10'	101.7 (5)	H9A—C9—H9B	107.8
C10—N3—C11	108.8 (4)	N3—C10—H10A	109.5
C11'—N3—C11	56.1 (4)	N3—C10—H10B	109.5
C10'—N3—C11	137.4 (4)	H10A—C10—H10B	109.5
C10—N3—C9'	138.0 (4)	N3—C10—H10C	109.5
C11'—N3—C9'	117.4 (4)	H10A—C10—H10C	109.5
C10'—N3—C9'	111.8 (5)	H10B—C10—H10C	109.5
C11—N3—C9'	63.6 (3)	N3—C11—H11A	109.5
C10—N3—C9	111.3 (3)	N3—C11—H11B	109.5
C11'—N3—C9	144.7 (4)	H11A—C11—H11B	109.5
C10'—N3—C9	71.2 (4)	N3—C11—H11C	109.5
C11—N3—C9	105.2 (4)	H11A—C11—H11C	109.5
C10—N3—Ni1	115.1 (3)	H11B—C11—H11C	109.5
C11'—N3—Ni1	111.9 (3)	N3—C9'—C8	112.8 (3)
C10'—N3—Ni1	109.0 (3)	N3—C9'—H9'A	109.0
C11—N3—Ni1	113.0 (3)	C8—C9'—H9'A	109.0
C9'—N3—Ni1	105.0 (3)	N3—C9'—H9'B	109.0
C9—N3—Ni1	102.9 (2)	C8—C9'—H9'B	109.0
C12—N4—Ni1	170.1 (3)	H9'A—C9'—H9'B	107.8
C13—N5—Ni1	158.1 (3)	N3—C10'—H10D	109.5
N1—C1—C2	121.8 (3)	N3—C10'—H10E	109.5
N1—C1—H1	119.1	H10D—C10'—H10E	109.5
C2—C1—H1	119.1	N3—C10'—H10F	109.5
C3—C2—C1	118.9 (3)	H10D—C10'—H10F	109.5
C3—C2—H2	120.5	H10E—C10'—H10F	109.5
C1—C2—H2	120.5	N3—C11'—H11D	109.5
C2—C3—C4	119.9 (3)	N3—C11'—H11E	109.5
C2—C3—H3	120.0	H11D—C11'—H11E	109.5
C4—C3—H3	120.0	N3—C11'—H11F	109.5
C3—C4—C5	118.3 (3)	H11D—C11'—H11F	109.5
C3—C4—H4	120.8	H11E—C11'—H11F	109.5
C5—C4—H4	120.8	N4—C12—S1	179.1 (3)
N1—C5—C4	121.9 (3)	N5—C13—S2	177.5 (3)

### Hydrogen-bond geometry (Å, °)

**Table:**

Cg1 is the centroid of the N1,C1–C5 ring.

**Table:**

*D*—H···*A*	*D*—H	H···*A*	*D*···*A*	*D*—H···*A*
O1—H1B···S1^i^	0.82 (2)	2.38 (2)	3.181 (3)	164 (4)
O1—H1A···S2^ii^	0.84 (2)	2.35 (2)	3.190 (3)	178 (4)
C7—H7C···Cg1^iii^	0.98	2.88	3.531 (3)	125

Symmetry codes: (i) *x*−1/2, −*y*+3/2, *z*−1/2; (ii) *x*−1/2, −*y*+3/2, *z*+1/2; (iii) *x*, −*y*+2, *z*−1/2.

## References

[bb1] Barbour, L. J. (2001). *J. Supramol. Chem.* **1**, 189–191.

[bb2] Bhowmik, P., Chattopadhyay, S., Drew, M. G. B., Diaz, D. & Ghosh, A. (2010). *Polyhedron*, **29**, 2637–2642.

[bb3] Bruker (2007). *APEX2* and *SAINT* Bruker AXS Inc., Madison, Wisconsin, USA.

[bb4] Diao, Y.-P. (2007). *Acta Cryst.* E**63**, m1453–m1454.

[bb5] Flack, H. D. (1983). *Acta Cryst.* A**39**, 876–881.

[bb6] Sheldrick, G. M. (1996). *SADABS* University of Göttingen, Germany.

[bb7] Sheldrick, G. M. (2008). *Acta Cryst.* A**64**, 112–122. 10.1107/S0108767307043930 18156677

[bb8] Suleiman Gwaram, N., Ikmal Hisham, N. A., Khaledi, H. & Mohd Ali, H. (2011). *Acta Cryst.* E**67**, m108. 10.1107/S1600536810052578 PMC3050266 21522521

[bb9] Westrip, S. P. (2010). *J. Appl. Cryst.* **43**, 920–925.

